# Impact of Microbial Dynamics During Composting on Product Quality and Soil Biological Enrichment Efficiency

**DOI:** 10.3390/microorganisms14061205

**Published:** 2026-05-27

**Authors:** Laura Núñez-Rodríguez, Marco Suárez-Estrada, Daniel Torres-Cuesta, Karen Polanía-Hincapié, Jose Moreno-Bermúdez, Lady Molano-Chávez, Juan Chavarro-Bermeo, German Estrada-Bonilla

**Affiliations:** 1 Colombian Agricultural Research Corporation (AGROSAVIA)—C.I. Tibaitatá, km 14 via Mosquera, Mosquera 250047, Colombia; lxnunez@agrosavia.co (L.N.-R.); msuarez@agrosavia.co (M.S.-E.); dtorres@agrosavia.co (D.T.-C.); tecnologia@amazoniaemprende.com (J.M.-B.); lmolano@agrosavia.co (L.M.-C.); jchavarro@agrosavia.co (J.C.-B.); 2 Department of Soil Science, Federal University of Lavras, Clover Roundabout Professor Edmir Sá Santos, Lavras 37203-202, MG, Brazil; kar.polania@udla.edu.co

**Keywords:** compost microbiome, nutrient cycling, nitrogen conservation, phosphorus bioavailability, organic amendments

## Abstract

Microbial communities regulate the transformation and stabilization of nutrients during composting; however, current knowledge on their specific functional roles across composting stages remains poorly integrated. This review examines the pivotal role of microbial mediation in nitrogen (N) and phosphorus (P) dynamics during composting and their subsequent impact on soil health. We analyze how biotechnological interventions—specifically the inoculation of functional microbial consortia (phosphate-solubilizing bacteria, phosphate-accumulating bacteria, and nitrifiers) and the application of physicochemical additives such as biochar—reconfigure microbial succession patterns to mitigate gaseous losses and enhance nutrient bioavailability. Several studies have reported substantial reductions in ammonia (NH_3_) and nitrous oxide (N_2_O) emissions under specific composting conditions, while simultaneously promoting the stabilization of labile P into more recalcitrant forms, including polyphosphates. Furthermore, the application of mature compost to agricultural systems induces a profound ecological reassembly of the soil microbiome, shifting community composition toward copiotrophic dominance (Pseudomonadota and Bacteroidota) and increasing functional redundancy. These microbial and functional shifts enhance soil resilience to environmental stressors—such as drought and temperature fluctuations—by stabilizing extracellular enzyme activity and reinforcing microbial co-occurrence networks. We conclude that managing microbial interactions along the compost–soil continuum is essential for developing organic amendments optimized for specific soil and crop requirements. This integrated approach represents a cornerstone of precision sustainable agriculture and contributes to climate change mitigation through soil health restoration.

## 1. Introduction

In intensive agricultural systems, the accumulation of organic waste has become a growing environmental problem due to its association with greenhouse gases emissions, unpleasant odors, eutrophication of aquatic ecosystems, groundwater contamination, and soil quality degradation [1,2]. In response to these challenges, composting has emerged as a biotechnological strategy for the valorization of residual biomass, enabling the controlled transformation of organic waste into amendments with high agronomic value. Beyond waste volume reduction and organic matter stabilization, composting produces organic fertilizer that improves soil structure, enhances water retention, and stimulates soil microbial biodiversity [3,4,5,6]. Consequently, composting occupies a central role within circular bioeconomy frameworks and sustainable agriculture models, particularly under conditions of production intensification, population growth, and structural limitations in waste management [7,8].

**Figure 1:**
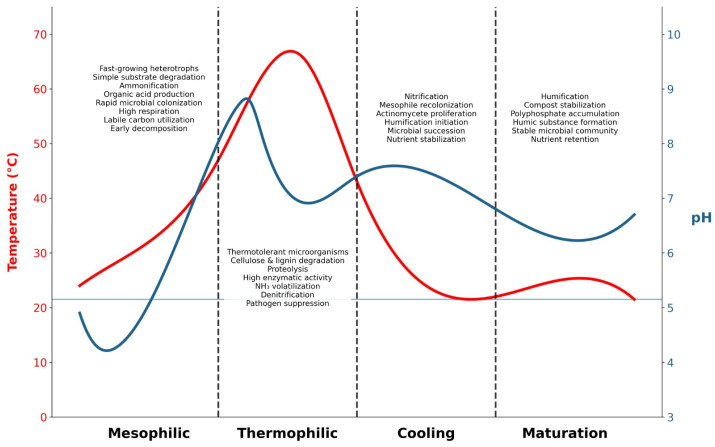
Schematic representation of the main composting phases, including temperature and pH dynamics and the predominant microbial functions associated with each stage of the process.

From a biological perspective, composting is an aerobic process of organic matter transformation mediated by microbial communities that utilize carbon (C) as an energy source and nitrogen (N) as a fundamental component of biomass synthesis and the production of extracellular enzymes [9,10]. Through coordinate hydrolysis, mineralization, oxidation, and humification reactions, microorganisms decompose both labile and recalcitrant substrates, release nutrients, generate metabolic heat, and promote the progressive formation of humic substances primarily humic acids (HA) and fulvic acids (FAs) which confer chemical and biological stability to the final product [11,12]. This process follows a thermal and ecological succession comprising mesophilic, thermophilic, cooling, and maturation phases (Figure 1), during which both the taxonomic composition of the microbiota and their dominant metabolic functions change. Early stages are dominated by copiotrophic microorganisms that rapidly exploit easily degradable substrates, whereas the thermophilic phase favors heat-tolerant taxa specialized in the intensive degradation of complex organic compounds and pathogen inactivation. During the maturation phase, mesophilic microorganisms and actinobacteria recolonize the compost and contribute to recalcitrant substrate degradation, humification, and product stabilization [13,14,15,16].

The efficiency and stability of these transformations depend closely on physicochemical parameters that regulate microbial metabolism. Temperature reflects the biological intensity of the system and controls phase transitions, while also defining the sanitization potential of the material [17]. pH modulates enzymatic activity, nutrient solubility, and processes such as ammonification, nitrification, and phosphorus (P) precipitation, remaining within optimal ranges (6.5–9.0) and stabilizing between 7.0 and 8.5, which confirmed compost maturity [17,18,19]. Moisture, ideally between 50 and 65%, is essential to sustain substrate transport and microbial activity; excessive values favor anaerobic conditions, whereas low values limit decomposition [17,18,19]. Likewise, C/N (25–30: 1) and C/P (135–160: 1) ratios regulate the relative availability of energy and nutrients for microbial growth, conditioning degradation rates [20,21,22], N retention, and P availability in the final product [23,24,25,26,27]. Aeration, in turn, sustains aerobic respiration and reduces the formation of anaerobic microenvironments associated with gaseous N losses, especially in the form of ammonia (NH_3_) and nitrous oxide (N_2_O) [13,28,29,30,31].

In this context, the microbiota constitutes the functional engine of composting. Bacteria, fungi, and, more recently, viral communities detected through metagenomic approaches participate differentially in organic matter depolymerization, nutrient mineralization and conservation, humic compound formation, and pathogen suppression [32,33,34,35,36]. Bacteria belonging to the phylum Bacillota, including genera such as *Bacillus* and *Geobacillus*, are essential due to their thermotolerance (>50 °C); beyond their lignocellulose degrading capacity, they synthesize proteases and pectinases while breaking down non-digestible carbohydrates like cellulose [37]. Within the Actinomycetota phylum, the genera *Streptomyces* and *Thermoactinomyces* stand out for being active in both mesophilic and thermophilic phases, effectively colonizing the substrate to decompose lignocellulose in plant tissues, as well as chitin and insect exoskeletons, through the production of extracellular enzymes such as α-amylase, glucoamylase, glucose isomerase, proteases, and lignin peroxidase [38]. Pseudomonadota constitutes the most abundant phylum detected at the onset of the composting process, functioning in the anaerobic degradation of organic matter and playing a critical role in the sulfur, nitrogen, and carbon cycles [39]. The role of the Chloroflexota phylum is crucial during the maturation phase; as chemolithotrophs or heterotrophs, they participate in biological nutrient removal and the biogeochemical chlorine cycle, ensuring the complete degradation of hemicellulose in mature compost [40]. Meanwhile, fungi specifically Ascomycota and Basidiomycota are the primary decomposers of plant tissues, particularly in the final stage when temperatures drop below 54 °C, finalizing the degradation of cellulose, xylan, and other recalcitrant compounds that were not fully processed by bacteria [41].

For a long time, knowledge of these communities was limited by culture-dependent methodologies, which were unable to adequately represent the complexity and functional succession of the active microbiota [42]. However, the development of high-throughput sequencing technologies, together with metagenomic tools and functional analyses, has made it possible to reveal with greater resolution the microbial mechanisms that regulate organic matter biotransformation, nutrient cycling, and compost maturation [27,33,34]. This mechanistic perspective has also supported targeted composting strategies based on the inoculation of functional consortia and the use of physicochemical additives to modulate microbial succession, reduce emissions, and improve nutrient conservation [17,19,43,44].

Beyond the composting process itself, the biological quality of compost has direct implications for soil functioning. Once applied, mature compost not only supplies organic C and nutrients, but also modifies the structure, diversity, and functionality of soil microbial communities, thereby promoting processes associated with fertility, ecological resilience, and soil health [45,46,47]. In this context, composting also acts as a biotechnological pathway for transforming N and P contained in organic residues into a stable, value-added product beneficial for soil health [48,49]. Both elements are tightly regulated by microbial activity, but their transformation pathways differ and are often addressed independently in the literature. Compost not only recycles P back into the agroecosystem, but, especially when combined with amendments such as biochar, can also increase pH, N and potassium contents, and microbial diversity, with positive effects on crop productivity [50,51]. Microbial N transformations, including ammonification, nitrification, denitrification, immobilization, and volatilization, determine both its conservation or loss during the process and its agronomic value in the final amendment Similarly, the dynamics and final bioavailability of P depend directly on the metabolic activity of the microbial community, since bacteria and fungi mediate shifts among different fractions through enzymatic and biogeochemical mechanisms. Therefore, understanding the coupled microbial regulation of N and P transformations is essential for improving nutrient use efficiency and reducing environmental impacts in compost-based systems.

Although numerous studies have addressed N or P dynamics during composting, existing reviews frequently adopt a fragmented perspective, focusing on individual nutrients or operational parameters while overlooking the microbial mechanisms that link waste transformation, compost quality, and the capacity of organic amendments to improve soil microbial activity and nutrient cycling. The primary contribution of this review is to provide an integrative perspective linking microbial processes involved in organic waste transformation, compost maturation, and soil microbial responses following compost application. By synthesizing current evidence, we highlight how microbial dynamics jointly regulate N and P transformations, compost maturation, emission mitigation, and the potential of organic amendments to biologically enrich soils. This integrative framework provides a conceptual basis for the development of tailored composting strategies aimed at maximizing nutrient recycling efficiency, enhancing soil health, and strengthening agroecosystem sustainability. Therefore, the objective of this review is to integrate current knowledge on microbial succession and nutrient transformation during composting and to discuss their implications for compost quality and soil microbial enrichment in agricultural systems.

## 2. Materials and Methods

For this literature review, an exhaustive search was conducted in the Web of Science “www.webofscience.com (accesed on 10 March 2026)” and Scopus “www.scopus.com (accesed on 10 March 2026)” databases. The initial literature selection was based on the following keywords: #1: “Composting” OR “Compost”; #2: “microbial community”; #3: “Rock Phosphate” OR “Phosphorus”; and #4: “Nitrogen” OR “Urea.” Additionally, a specific search was performed to address studies related to the application of organic amendments and fertilizers to the soil. Search results were integrated using the Boolean operator “AND.” Subsequently, the retrieved articles were filtered to exclude those of limited relevance, prioritizing studies that specifically addressed microbial dynamics during the composting process and the role of organic amendments—particularly compost—in modulating soil microbiology.

## 3. The Role of Microbial Succession in Composting Process

**Figure 2:**
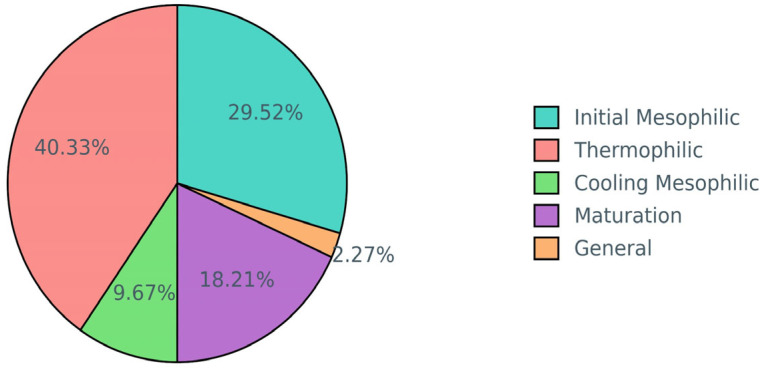
Percentage distribution of microbial taxa reported across the different composting phases. The figure illustrates the relative proportion of microbial taxa reported in the literature for each composting phase, calculated based on the frequency of occurrence across the studies included in this review. Percentages reflect reported taxonomic presence rather than absolute microbial abundance.

Microorganisms constitute the biological engine of the composting process, which unfolds through a sequence of thermally defined phases that act as ecological filters driving microbial succession. As illustrated in Figure 2, the highest proportion of microbial taxa identified across the reviewed studies was concentrated in the thermophilic (40.3%) and initial mesophilic (29.5%) phases—stages characterized by intense metabolic activity and rapid organic matter transformation [35]. The greater number of taxa reported during these phases likely reflects the strong physicochemical changes and the wide range of microbial functions associated with the degradation of easily available substrates, carbon mineralization, and N transformations [44]. In practical terms, the thermophilic phase represents one of the most critical stages of composting because it promotes rapid organic matter decomposition, accelerates nutrient turnover, and contributes to pathogen and weed seed suppression through sustained high temperatures. These processes are essential for producing a biologically stable and sanitized compost suitable for agricultural applications. In contrast, the cooling and maturation phases showed a lower proportion of taxa across the analyzed studies, which may be associated with substrate depletion and the progressive stabilization of the composting matrix [35]. This transition suggests a shift from highly active decomposer communities toward more specialized microorganisms involved in humification and nutrient stabilization processes. Consequently, microbial succession throughout composting directly influences compost maturity, stability, nutrient availability, and phytotoxicity reduction, thereby determining the agronomic quality of the final product and its potential contribution to sustainable soil management [9,10].

**Figure 3:**
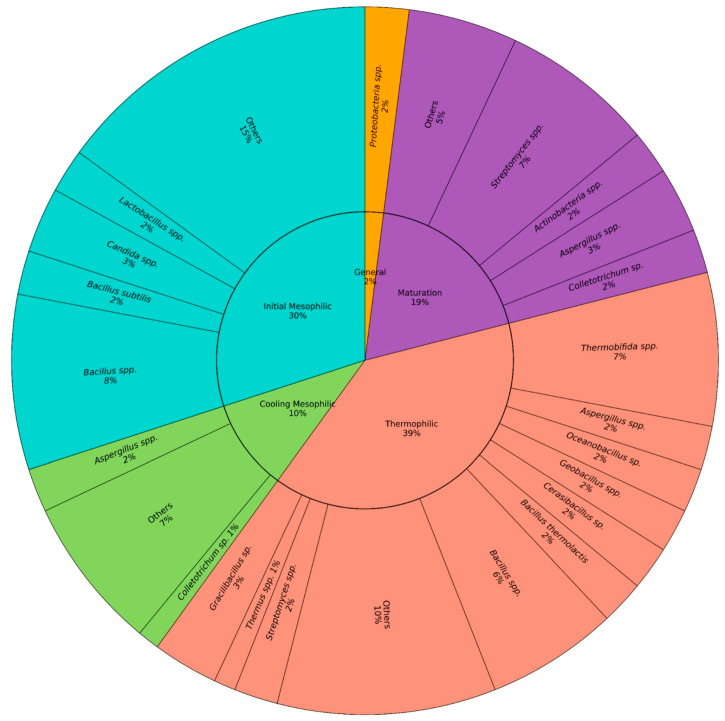
Principal microbial taxa reported throughout the different phases of the composting process. The figure summarizes the dominant bacterial, fungal, and viral taxa associated with each composting phase based on the studies included in this review.

**Figure 4:**
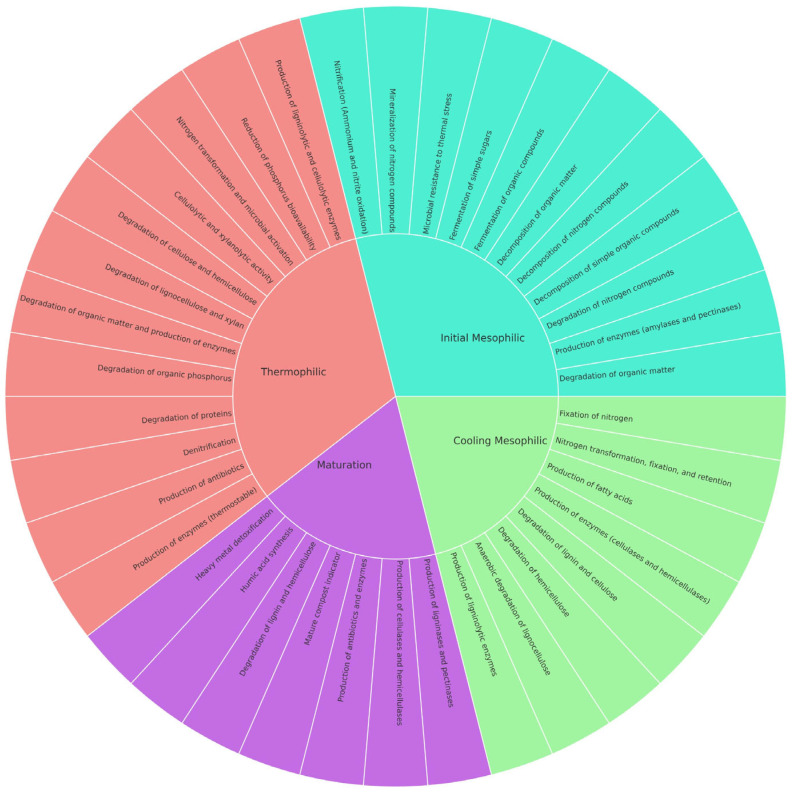
Principal potential microbial functions associated with the different phases of the composting process. The figure illustrates the dominant microbial functions inferred from taxonomic composition and functional gene evidence reported in the literature, including organic matter degradation, nutrient transformations, humification, and pathogen suppression. Functions represent potential activities associated with each phase rather than direct measurements.

Microbial succession during composting involves both taxonomic and functional transitions that are tightly coupled to the thermal dynamics of the process (Figure 3 and Figure 4). Early stages are typically dominated by fast-growing microorganisms responsible for the degradation of readily biodegradable substrates and the initiation of N transformations. As composting progresses into the thermophilic phase, microbial communities reorganize toward thermotolerant taxa capable of sustaining metabolic activity under elevated temperatures. These microorganisms produce thermostable enzymes that enable the efficient degradation of complex organic compounds, including cellulose, hemicellulose, proteins, and lipids, while simultaneously contributing to pathogen inactivation [27,52,53].

During the cooling and maturation phases, the gradual decline in temperature allows the recolonization of microbial groups associated with nutrient stabilization and humification. At this stage, nitrifying microorganisms contribute to N stabilization through the oxidation of ammonium (NH_4_^+^), while bacteria and fungi specialized in the degradation of recalcitrant compounds promote the transformation of complex organic matter and the formation of humic substances [11,19,44]. These later phases are therefore critical for determining compost maturity, stability, and agronomic quality.

Recent metagenomic studies have expanded the understanding of composting microbiomes by revealing structured viral communities. In particular, bacteriophages belonging to the phylum Uroviricota are associated with microbial lysis and internal nutrient recycling, contributing to the secondary release of C and N during the process [27,36]. Increased phage prevalence has been linked to microbial processes that favor the accumulation of microbially derived dissolved organic matter in mature compost, highlighting viruses as emerging regulators of microbial turnover and organic matter transformation [54].

The dynamic interaction between composting thermal phases and microbial community structure results in a well-defined ecological succession. To synthesize these interactions, Table 1 summarizes the taxonomic shifts and functional transitions reported across the different composting stages, highlighting the complementary roles of bacteria, fungi, and viral communities in organic matter decomposition, nutrient cycling, and compost stabilization.

## 4. Microbial Mediation of Nitrogen Dynamics During Composting: Transformation Mechanisms and Control Factors

Ammonification represents the first fundamental step in the N cycle during composting, whereby organic N is converted into NH_4_^+^ by heterotrophic microorganisms, primarily bacteria and fungi [59]. This process is particularly intense during the early stages of composting, peaking reaching maximum rates during the initial mesophilic and thermophilic phases, when substrate availability and microbial activity are highest [60]. Among the most relevant groups in the ammonification process are members of the phyla Bacillota, Actinomycetota, and Pseudomonadota, which include key genera such as *Bacillus* spp., *Anaerococcus* spp., and *Pseudogracilibacillus* spp. These taxa are characterized by a high ammonifying capacity derived from their ability to produce proteolytic and chitinolytic enzymes [27,61]. During the thermophilic phase, thermotolerant bacteria such as *Pseudogracilibacillus* spp. and *Oceanobacillus* spp. dominate ammonification processes due to their resistance to elevated temperature [27]. These bacteria secrete hydrolytic enzymes that degrade complex organic compounds into simpler molecules, facilitating the release of mineral N [32]. However, the concomitant increase in temperature and pH during this phase enhances the conversion of NH_4_^+^ into NH_3_, promoting volatilization losses that constitute one of the primary pathways of N loss during composting [62,63].

When released NH_4_^+^ exceeds microbial assimilation capacity, N is lost primarily through NH_3_ volatilization [64]. Microbial immobilization processes can partially counterbalance these losses by incorporating NH_4_^+^ into microbial biomass, thereby competing with NH_3_ emission pathways [62,65]. In parallel, oxidation through nitrification, a two-step process mediated by ammonia-oxidizing bacteria (AOB), such as *Nitrosomonas* spp., which oxidize NH_4_^+^ to nitrite (NO_2_^−^), and nitrite-oxidizing bacteria, such as *Nitrobacter* spp., which further oxidize NO_2_^−^ to nitrate (NO_3_^−^) [31,53]. Nitrification activity is strongly inhibited during the thermophilic phase by temperatures exceeding 40 °C and high NH_4_^+^ concentrations [60]. Consequently, this process predominantly occurs during the cooling and maturation phases, when environmental conditions become favorable for nitrifier recolonization [11].

In addition to nitrification, denitrification represents N loss pathway during composting. Under anaerobic or microaerophilic conditions, denitrifying microorganisms reduce nitrate and nitrite to gaseous N species, including nitric oxide (NO), N_2_O, and dinitrogen (N_2_) [66,67,68]. Denitrifying bacteria carrying the key denitrification genes, such as *nirK* and *nirS*, belonging to genera such as *Luteimonas* spp., *Achromobacter* spp., and *Alcaligenes faecalis*, are particularly important contributors to N losses during the process [31]. Denitrification activity is favored in poorly aerated zones and contributes significantly to greenhouse gas emissions associated with composting systems [69].

Nitrogen fixation also occurs during composting, primarily during the thermophilic and cooling phases, through the activity of diazotrophic microorganisms [70]. Among these, *Thermoclostridium stercorarium* (phylum Bacillota) has been identified as a key N-fixing species capable of increasing total N content in the final compost product [17,31]. Additionally, coupled denitrification pathways may directly reduce NO_2_^−^ to N_2_, further influencing N balance [71,72]. Collectively, the interplay among ammonification, nitrification, denitrification, immobilization, and biological fixation ultimately determines whether composting systems function as N-conserving or N-losing processes [34]. Nitrogen losses not only reduces process efficiency but also compromises the agronomic quality, maturity, and environmental sustainability of compost [69,73]. These N excessive losses can alter heterotrophic microbial activity, which reduces biodegradation efficiency and delay compost stabilization [6,16].

### Strategies for Nitrogen Modulation in Composting Systems

Nitrogen dynamics during composting are fundamentally governed by microbial interactions that regulate ammonification, nitrification, denitrification, and immobilization processes. In recent years, a range of biotechnological strategies has been proposed to modulate these microbial pathways with the objective of mitigating gaseous N losses, enhancing N retention, and improving the agronomic quality of the final compost. Among these strategies, microbial inoculation and the application of physicochemical additives have received particular attention. Accumulating evidence indicates that microbial inoculants and physicochemical additives operate through complementary mechanisms that reshape microbial succession and regulate N transformation pathways. The inoculation of functional microbial consortia enhances enzymatic activity and selectively promotes functional groups involved in ammonification, nitrification, and N fixation, thereby favoring N conservation and reducing gaseous losses [12,17,44]. In parallel, physicochemical additives modify the composting microenvironment by improving aeration, buffering pH, increasing water-holding capacity, and enhancing NH_4_^+^ retention, collectively contribute to the mitigation of NH_3_ and N_2_O emissions [74,75,76].

Synergistic interactions between biological and structural strategies have been shown to further enhance N conservation by stabilizing microbial communities and redirecting N cycling toward more conservative pathways [77,78]. These interventions promote the retention of N in mineral and organic forms, increase microbial biomass N, and improve humification process [79]. Current evidence suggests that these strategies operate through complementary mechanisms that reshape both substrate properties and microbial ecology, thereby enhancing N retention, reducing gaseous emissions, and improving compost stability.

Table 2 integrates the main biotechnological approaches, emphasizing their role in regulating N transformations and promoting N conservation during composting. The effectiveness of these strategies significantly depends on both the specific biochemical mechanism of the additive and the operational parameters applied. While porous materials like clinoptilolite zeolite rely on high cation exchange capacity for the physical sequestration of NH_4_^+^ [80], emerging technologies like Iron Oxide Nanoparticles (FeONPs) directly modulate microbial ecology. Specifically, α-Fe_2_O_3_ and Fe_3_O_4_ nanoparticles (10 mg kg^−1^) have been shown to reduce total nitrogen losses by selectively inhibiting Ammonia-Oxidizing Bacteria (AOB) while maintaining Ammonia-Oxidizing Archaea (AOA) activity. Aeration management also remains a critical factor; increasing free air space from 45% to 65% has been reported to reduce NH_3_ emissions by 32%, while intermediate levels (55%) favor nitrifier proliferation [81]. The impact of biological agents further underscores this complexity; for instance, in swine manure, microbial inoculation enhances enzyme activities to increase mineral N contents [82]. Similarly, in chicken manure composting, the combined application of a carbon-based microbial agent and a biotrickling filter has been shown to accelerate pile heating and achieve NH_3_ removal rates exceeding 90% [36]. Finally, chemical additives like selenite [Se (IV)] exhibit distinct dose-dependent and multi-gas responses. In goat manure systems, Se (IV) supplementation reduced NH_3_ emissions by 3.5–42.4%, whereas significant reductions in N_2_O emissions—reaching up to 69.54%—were primarily observed at higher dosages (8–10 mg kg^−1^) [83]. Collectively, these studies highlight that mitigation efficiencies are highly context-dependent, requiring a precise alignment between additive mechanisms and operational conditions.

## 5. Microbial Mediation of Phosphorus Dynamics During Composting: Transformation Mechanisms and Control Factors

The total P content of finished compost is an insufficient indicator of its agronomic value, as plant and microbial uptake is governed primarily by P bioavailability than total concentration. Phosphorus bioavailability is determined by its chemical speciation, which is dynamically modified throughout the composting process under the combined influence of microbial activity and physicochemical conditions [89]. During composting, P continuously transitions between organic P (Po) and inorganic P (Pi) fractions, with transformation pathways tightly regulated by decomposition processes, enzymatic activity and environmental variables.

As organic matter decomposes, P is progressively released and incorporated into evolving organic matrices, particularly humic substances mainly HA and FA. These compounds play a dual role in P dynamics [76]. On one hand, their functional groups, including carboxyl and hydroxyl moieties, act as effective chelating agents capable of complexing cations such as Ca^2+^, Fe^3+^, and Al^3+^, thereby preventing phosphate and enhancing P solubility [90]. Conversely, humic substances can promote controlled P immobilization through the formation of stable P–humic complexes, which protect phosphate from mineral adsorption and leaching losses [76]. Exist evidence of negative correlation between humification and P bioavailability, based on metal bridging or chelation that promotes the biological fixation of [16,44]. The degree of humification, commonly expressed as the HA/FA ratio, therefore emerges as a critical indicator of P stabilization during compost maturation [43].

Phosphorus availability during composting is inherently dynamic and biologically mediated, as summarized by the diverse P fractions reported (Table 3). While Pi fractions are strongly influenced by pH, redox conditions, and cation concentration, the transformation of recalcitrant Po compounds—particularly phytates and phosphonates—into bioavailable orthophosphate is strictly dependent on microbial metabolism. This conversion is driven by the secretion of specific extracellular enzymes, such as phosphomonoesterases, phytases, and C–P lyases, which enable microorganisms to access and mineralize otherwise inaccessible Po. Consequently, the succession and functional capacity of microbial communities represent key determinants of P-cycling efficiency throughout the composting process.

**Table 3:** Classification of inorganic and organic phosphorus forms and their relative bioavailability during composting.

Category	Phosphorus Form	Bioavailability	Key References
**Inorganic P (Pi)**	**Exchangeable P**	**High.** Most labile fraction, weakly adsorbed, and immediately available for plant uptake.	[43]
	**Metal-bound P (Al-P, Fe-P, Ca-P)**	**Moderate to Low.** Solubility is pH-dependent; Al-P and Fe-P are stable in acidic conditions, while Ca-P dominates in alkaline environments.	[19,43,90]
	**Struvite**	**Moderately available.** Crystalline P form formed under Mg^2+^-rich and slightly acidic conditions.	[90]
	**Occluded P**	**Very Low.** Recalcitrant and sequestered within stable mineral matrices; generally unavailable.	[19,43]
**Organic P (Po)**	**Phosphate Esters (incl. Phytates)**	**Moderate to low.** Includes DNA, RNA, and phospholipids. Phytates are highly resistant and require phytase enzymes for P release.	[23,49,90,91]
	**Polyphosphates (Poly-P)**	**Moderate.** Accumulated by microorganisms as a slow-release reservoir	[16,90,92]
	**Phosphonates**	**Low.** Contains stable C-P bonds; requires specialized C-P lyase enzymes for cleavage.	[43]

### 5.1. Molecular Mechanisms of Microbial Phosphorus Transformation

The mineralization of Po during composting is fundamentally mediated by extracellular enzymes synthesized and secreted by microorganisms. Among these, Phosphomonoesterases—both acid (AcP) and alkaline (AlP)— play a dominant role by hydrolyzing phosphoester bonds in compounds such as phospholipids, nucleotides and sugar phosphates [49,91]. The activity of these enzymes is strongly influenced by substrates availability and pH [25]. Phosphomonoesterase activity is frequently stimulated under conditions of low P availability (high C/P ratios) [25] and can be enhanced through interventions such as the inoculation of phosphate-solubilizing bacteria (PSB) inoculation [49] or the application of microbial fuel cell (MFC) [43]. In contrast, high concentrations of readily available P can suppress enzyme production through feedback inhibition [25]. The abundance of *phoD* gene, encoding alkaline phosphomonoesterase, is used as a molecular biomarker of microbial P mineralization potential [72,91], and is closely linked to the activity of broader C, N, P, and S cycling processes [91].

Phytases constitute a specialized class of enzymes responsible for the hydrolysis of phytate (myo-inositol hexakisphosphate), one of the most abundant forms of Po in plant-derived residues [43,49,91]. Their activity is particularly relevant during the composting of crop residues and agroindustrial wastes rich in phytate-bound P [49]. In addition, C-P lyases play a crucial role in the degradation of phosphonates by cleaving stable C–P bonds, thereby releasing phosphate from otherwise persistent organic compounds [43].

In parallel with mineralization pathways, phosphate solubilization represents a key mechanism regulating Pi availability. Phosphate-solubilizing microorganisms (both bacteria and fungi) excrete low-molecular-weight organic acids such as citric, gluconic, oxalic, lactic, succinic, acetic, and formic acids [48]. These metabolites promote P solubilization through two complementary mechanisms: acidification of the microenvironment via protons (H^+^) release, which enhances the dissolution of calcium phosphates [48], and chelation of metal cations, which liberates phosphate from Al-, Fe-, and Ca-bound forms [90]. The *pqqC* gene, associated with gluconic acid biosynthesis, is therefore considered an important functional marker for microbial Pi solubilization potential [23].

In contrast to solubilization, phosphate-accumulating bacteria (PAB) contribute to P immobilization and stabilization by assimilating soluble phosphate and storing it intracellularly as polyphosphate (Poly-P). This process is regulated by the polyphosphate kinase (*ppk*) gene and represents an adaptive microbial strategy for P storage under fluctuating environmental conditions. Inoculation with PAB has been shown increase microbial biomass P and favor the formation of slow-release P pools, thereby reducing the risk of P losses during compost application [92]. Secondary Mechanisms, including the production of siderophores and exopolysaccharides further contribute to P mobilization by modulating metal availability and enhancing microbial access to Pi pools [90].

### 5.2. Strategies for Phosphorus Modulation in Composting Systems

Phosphorus transformation during composting arises from the coordinated activity of complex microbial networks rather than the action of isolated functional groups. The process typically follows a sequential cascade in which primary decomposers initiate organic matter breakdown, thereby liberating Po compounds embedded within complex plant polymer [93]. These bacteria secrete enzymes like cellulases to break down the structural biopolymers of plant matter [93]. Only after this initial depolymerization can specialized microorganisms—such as phosphate-solubilizing and mineralizing taxa—access and transform distinct P substrates through solubilization, immobilization, or enzymatic mineralization pathways [93].

Throughout composting, solubilization (increase in Pi availability), immobilization (P sequestration), and mineralization (conversion of Po to Pi) occur simultaneously, with the net balance among these processes determining final P bioavailability [94,95]. Because these transformations are biologically regulated, shifts in microbial community composition, functional gene abundance, and enzymatic activity exert strong control over the direction and magnitude of P fluxes [96]. Functional groups such as PSB, including genera such as *Bacillus*, *Pseudomonas*, *Enterobacter*, *Rhizobium*, *Aspergillus,* and *Penicillium* among others play central role in mobilizing insoluble P through organic acid production and enzymatic activity [19,23,48,95,97,98,99,100].

Phosphate-accumulating bacteria represent an additional functional group capable of buffering excess soluble P through intracellularly Poly-P storage, thereby converting labile P into stabilized, slow-release forms [92]. In this context, the genus *Halocella* spp. has been identified as a keystone taxon associated with the Poly-P accumulation pathway [92].

The modulation of these microbial pathways can be achieved through targeted interventions, including microbial inoculation, carbon-based amendments, mineral additives, and emerging technologies. As summarized in Table 4, these strategies enable the orientation of P transformations toward either enhanced bioavailability or increased stabilization, depending on agronomic objectives and environmental constraints.

## 6. Soil Microbial Modulation Associated with Organic Amendments in Productive Systems: A Compost-Centered Perspective

Compost represents one of the most extensively studied organic amendments for modulating soil microbial communities and ecosystem functioning. Although other organic amendments such as manure, vermicompost, and biochar can induce related biological responses, compost differs due to its stabilized organic matter, partially humified composition, and complex microbiological structure generated during the composting process [105,106]. These characteristics allow compost to influence soil biology by altering resource availability, trophic interactions, and the physicochemical conditions of the edaphic environment [105,107], thereby promoting niche diversification, activating critical metabolic pathways, and enhancing essential ecological processes such as nutrient cycling [69,108]. The application of organic amendments modifies soil parameters such including pH, moisture, organic C content, aeration, and the C/N ratio, creating heterogeneous microhabitats that shape microbial community structure, diversity, and functional activity [105,109].

These habitat modifications have direct effects on microbial processes involved in biogeochemical cycles, particularly those associated with C, N and P transformations. Organic amendments stimulate microbial processes such as mineralization, nitrification, denitrification, biological N fixation, and organic matter stabilization, while simultaneously influencing extracellular enzyme production and activity [108,110,111,112,113]. By accelerating successional dynamics, organic inputs initially favor microbial communities specialized in the degradation of labile substrates and subsequently promote consortia associated with humification and long-term C stabilization. This temporal reorganization of microbial communities reshape soil trophic networks and enhances metabolic efficiency, functional redundancy, and resilience [107,114].

In addition to serving as nutrient and energy sources, organic amendments act as multifunctional ecological modulators by providing physical surfaces and redox microenvironments that support the establishment of specific microbial assemblages. These mechanisms collectively regulate the availability of inorganic N forms and bioavailable P, stimulate enzymatic activities, and contribute to improved nutrient retention and use efficiency [108,111,115,116].

Beyond their effects on individual taxa or functions, organic amendments profoundly influence the architecture of microbial co-occurrence networks [117]. By reorganizing interactions among functional microbial groups, compost-based amendments increase network complexity and connectivity, attributes that are increasingly recognized as indicators of ecosystem stability and functional resilience [16,118].

Overall, current evidence suggests that the interaction between compost and soil microbial communities is regulated by the combined influence of the characteristics of the amendment, soil properties, and management practices. Compost maturity, organic matter composition, nutrient content, and application rate determine substrate quality and microbial colonization dynamics [21,119,120]. Simultaneously, soil properties such as pH, texture, mineralogy, moisture, and native microbial diversity regulate microbial establishment, nutrient accessibility, and functionality [45,121,122,123,124]. Furthermore, management practices, including tillage intensity, irrigation, fertilization regimes, and the frequency of soil amendment application, modify soil aeration, resource distribution, and habitat heterogeneity, thereby influencing microbial succession, the organization of co-occurrence networks, and ecosystem functioning [45,125,126,127]. This complexity highlights the need to adopt an integrative, microbiome-centered perspective when evaluating compost-based strategies in sustainable agricultural systems.

### 6.1. Reconfiguration of Soil Microbial Communities Mediated by Compost Application

The transition toward a biologically stable and functionally resilient soil depends largely on the capacity of compost to act simultaneously as a vehicle for microbial inoculation and as a modifier of the edaphic habitat. This dual role facilitates the colonization of bacteria and fungi within soil micro- and macroaggregates, promoting the establishment of metabolically active and functionally diverse microbial communities [13]. The addition of compost as an organic amendment consistently induces changes in soil microbial biomass, enzymatic activity, α-diversity (richness and evenness), and β-diversity (community composition) across both bacteria and fungi domains [128,129,130]. Organic compounds supplied by compost provide C and nutrient sources that support the establishment of heterogeneous microbial assemblages [131,132].

The input of organic C with varying degrees of chemical complexity promotes the coexistence of microorganisms with contrasting metabolic strategies, favoring more diverse and functionally redundant communities [133,134]. This increased functional redundancy enhances the stability of key ecological processes such as nutrient cycling, pathogen suppression, and organic matter turnover, thereby strengthening soil resilience to environmental perturbations [129,130,133,134,135]. Numerous studies have reported consistent increases in bacterial and fungal α-diversity following compost incorporation, largely attributed to improved substrate availability, nutrient supply, and microhabitat heterogeneity that alleviate resource limitations and competitive exclusion [13,47,129,130,132,136,137].

Compost application also drives pronounced shifts in β-diversity, reflecting a reassembly of the soil microbiome mediated by niche modification [129,138]. Regarding bacterial level, compost-amended soils typically exhibit increased relative abundance of copiotrophic phyla as Pseudomonadota, Bacteroidetes, Actinomycetota, and Bacillota, which are associated with high metabolic capacity and efficient degradation of labile organic matter. In contrast, the relative dominance of oligotrophic groups such as Acidobacteria is often reduced [46,108,113,133,136]. At the fungal level, compost incorporation commonly enhances the abundance of Ascomycota and Mortierellomycota, phyla closely linked to residue decomposition and nutrient mobilization in agriculture soils [45,78,129]. In contrast, Basidiomycota may decline under conditions of frequent compost applications due to reduced reliance on advanced ligninolytic degradation [45,78].

From a functional perspective, compost amendment generally increases microbial biomass C and stimulates enzymatic activities associated with C, N, and P cycling. These responses arise from both enhanced microbial synthesis and the physical stabilization of extracellular enzymes within organo-mineral complexes [111,139]. As a result, elevated activities of *β*-glucosidase, urease, and phosphomonoesterases are frequently observed, serving as indicators of an increased capacity for substrate depolymerization and nutrient turnover [107,113,140]. However, the magnitude and direction of these functional responses are strongly context-dependent, being influenced by compost maturity, application rate, soil physicochemical properties, and management history [132,140,141,142].

Overall, compost induced reconfiguration of the soil microbial communities leads to the formation of more complex and interconnected microbial networks with higher functional redundancy. These network-level changes enhance the stability of ecosystem functions over time and improve soil capacity to maintain productivity and biological activity under fluctuating environmental conditions [129,130,138,143]. Consequently, compost application emerges not only as a nutrient management practice but also as a powerful ecological driver of soil microbiome reassembly and functional resilience.

The observed changes in soil biological properties are closely linked to specific parameters of the organic amendment’s quality and stability. Compost maturity and its degree of humification, as reflected in the humic acid/fulvic acid ratio, modulate the structure of the microbial community by providing stable polymeric cores and more recalcitrant forms of carbon; this limits the proliferation of opportunistic taxa and promotes a sustained increase in microbial biomass and soil diversity, consolidating persistent taxa that can act as hubs within the edaphic ecosystem [21,144,145]. Additionally, a balanced C/N ratio in the amendment, close to optimal values of 15–20, reduces nitrogen immobilization processes and directly stimulates soil enzymatic activity, including cellulases, ureases, and phosphatases, associated with carbon and nutrient cycles [146,147,148]. Furthermore, nutrient speciation and the intrinsic chemical properties of compost, such as pH and salinity, regulate ionic solubility in the soil solution; these factors act as environmental filters that reshape biological interactions and determine whether the development of non-random patterns in the complexity of soil microbial co-occurrence networks is favored [21,45,122,124]. Finally, a low load of contaminants, such as heavy metals or persistent organic compounds, reduces direct ecotoxic effects on the microbiota, allowing interactions between bacteria and fungi to achieve greater interconnectivity, stability, and ecological resilience in complex microbial networks over the long term [21,145].

### 6.2. Experimental Evidence of Compost Effects on Soil Microbial Structure and Functionality

A growing body of experimental evidence shows that compost application leads to profound and lasting changes in the structure and function of soil microbial communities across a wide range of agroecosystems. Both short- and long-term field and mesocosm studies consistently show that compost amendments promote microbial reassembly by increasing biomass, diversity, and functional potential compared to mineral fertilization or controls without amendments. These responses are driven by the combined effects of organic C inputs, nutrient availability, and habitat modification.

Clear temporal shifts in soil microbial community composition following compost application compared with conventional fertilization have been observed [119]. Their results showed an early increase in α-diversity accompanied by enrichment of copiotrophic bacterial phyla, such as Bacteroidota, Bacillota, and Pseudomonadota. Additionally, the increased abundance of bacterial orders such as Cytophagales and Myxococcales, which are associated with particulate organic matter degradation and bacterial trophic regulation, indicated enhanced C recycling efficiency in compost -amended soils. These findings are consistent with long-term studies in Mediterranean agroecosystems, where poultry manure compost increased total microbial biomass and contributed to aggregate stability and C storage over multi-year periods compared to mineral fertilization [143].

Compost inputs provide chemically diverse C substrates that sustain higher microbial biomass and stimulate the production of microbial exudates and extracellular polymers. These compounds play a critical role in soil aggregation, reinforcing the formation of microhabitats that support spatially structured and metabolically active microbial communities [105,143,149]. Experimental evidence further indicates that compost-amended soils exhibit an enhanced capacity to degrade aromatic and complex, a key function linked to the formation of stable soil organic matter and long-term C sequestration.

Long-term compost applications have also been shown to improve crop productivity while reshaping rhizosphere microbial communities toward functionally beneficial assemblages. For example, sustained manure compost inputs (over 9 years) increased bacterial diversity and enriched taxa associated with plant growth promotion, including *Pseudomonas*, *Burkholderia*, and *Chrysosporium*, while simultaneously reducing the relative abundance of phytopathogenic fungi such as *Fusarium* [113]. Network analyses in these systems revealed increased co-occurrence complexity and the emergence of functional modules, suggesting intensified microbial interactions related to nutrient mobilization, antagonistic, and mutualistic. Positive correlation between beneficial microbial taxa and crop yield or quality metrics further reinforce the link between compost-induced microbial reassembly and agronomic performance [113].

Beyond productivity effects, compost amendments enhance soil functional stability under environmental stress. Studies conducted under drought conditions have shown that compost-amended soils maintain higher microbial biomass and enzymatic activity, particularly β-glucosidase and urease, compared to unamended soils. These responses are often accompanied by a shift toward copiotrophic bacterial and fungal taxa capable of rapidly exploiting available organic substrates, thereby sustaining metabolic activity under reduced moisture availability [130,148].

Temperature stress experiments further demonstrate that compost-amended soils exhibit lower sensitivity to abrupt thermal fluctuations than soils receiving mineral fertilizers. This increased resistance is attributed to the stabilization of extracellular enzymes within organo-mineral complexes and to the selection of microbial communities with metabolic strategies adapted to heterogeneous and resource-rich environments [106,150,151,152]. Collectively, these findings indicate that compost application not only enhances microbial diversity and function but also strengthens soil resilience by promoting microbial communities capable of maintaining ecosystem processes under variable climatic conditions.

Overall, experimental evidence confirms that compost acts as a powerful driver of soil microbial reassembly, promoting increases in biomass, diversity, network complexity, and functional redundancy. These microbiome-level changes translate into improved nutrient cycling, C stabilization, pathogen suppression, and crop performance, underscoring the role of compost as a multifunctional tool for enhancing soil health and ecosystem resilience in productive agricultural systems.

## 7. Conclusions and Future Perspectives

The evidence synthesized in this review demonstrates that composting, when strategically managed through biotechnological interventions, transcends its traditional role as a waste stabilization process to become a precision tool for soil restoration and sustainable nutrient management. Microbial communities emerge as the central drivers governing organic matter transformation, nutrient conservation, and the agronomic and ecological quality of the final compost product.

Microbially mediated nutrient conservation represents a key outcome of engineered composting systems. The inoculation of functional microbial consortia, combined with the application of porous additives such as biochar, optimizes N retention by accelerating the transition to thermophilic conditions, synchronizing enzymatic activities, and promoting NH_4_^+^ stabilization within organic and mineral matrices. These strategies effectively reduce N losses as NH_3_ and N_2_O, thereby improving compost maturity and reducing the environmental footprint of the process.

Microorganisms play a pivotal role in regulating the transformation of both Pi and Po pools through the coordinated action of extracellular enzymes, organic acid secretion, and intracellular polyphosphate accumulation. The functional interplay between PSB and PAB is central to shaping P dynamics during composting, governing the balance between P mobilization and stabilization processes. These biologically stabilized P forms reduce post-application losses and enhance nutrient use efficiency in agricultural systems.

Beyond nutrient management, the application of mature compost exerts a profound influence on soil microbial community reassembly. Compost functions not only as a source of C and nutrients but also as a biological inoculum and habitat modifier that shifts soil microbial communities toward copiotrophic dominance, increases functional redundancy, and strengthens microbial co-occurrence networks. These changes enhance soil resilience to environmental stressors, including drought and temperature fluctuations, by stabilizing extracellular enzyme activity and maintaining ecosystem functions under adverse conditions.

From a broader perspective, composting can be conceptualized as a microbial engineering platform capable of generating “tailor-made” organic amendments aligned with specific soil constraints, crop demands, and environmental objectives. However, important research gaps remain. Future studies should prioritize longitudinal, multi-omics approaches—particularly metatranscriptomics and metaproteomics—to track the real-time expression of N- and P-cycling genes as microbial communities transition from composting systems to soil and rhizosphere environments. There is an urgent need to integrate multi-omics data with high-resolution chemical speciation (e.g., Nuclear Magnetic Resonance, Stable Isotope Probing or synchrotron-based techniques) to unravel the exact molecular mechanisms behind organo-mineral complex formation and nutrient stabilization.

Additionally, future research should integrate compost microbiome engineering with field-scale validation, considering soil mineralogy, climate variability, and management practices. Expanding the scope of investigation to include the fate of emerging contaminants—such as microplastics, antibiotic resistance genes, and residual pharmaceuticals—will be critical to ensure the environmental safety of advanced composting strategies.

Ultimately, advancing toward precision composting will require a paradigm shift in which microbial ecology informs the rational design of organic amendments. By aligning microbial functionality with agronomic and environmental goals, composting can play a pivotal role in restoring soil health, enhancing agroecosystem resilience, and supporting climate-smart and sustainable agriculture.

## Figures and Tables

**Table 1:** Microbial succession and predominant functional activities across composting phases.

Phase	Temp. Regime	Key Bacterial Taxa	Key Taxa	Predominant Functions and Processes	References
**Initial Mesophilic**	Moderate (~25–45 °C)	*Bacillus* spp., *Bacillus subtilis*, *Lactobacillus* spp. (Bacillota); *Pseudomonadota*	*Candida* spp. (Ascomycota)	Degradation of labile organic matter; enzyme/hydrolase production; fermentation; early nitrification.	[35,44]
**Thermophilic**	High (>45–70 °C)	*Thermobifida* spp. (Actinomycetota); *Bacillus* spp., *Bacillus thermolactis*, *Geobacillus* spp., *Oceanobacillus* sp. (Bacillota)	Uroviricota (Bacteriophages)	Intensive cellulose degradation; thermostable enzyme secretion (proteases/lipases); ammonification; denitrification; microbial lysis.	[27,36,52,53,55,56]
**Cooling and Maturation**	Decreasing to Ambient	*Nitrosomonas* spp., *Nitrobacter* spp., *Streptomyces* spp. (Actinomycetota); *Virgibacillus* spp., *Gracilibacillus* spp.	*Aspergillus* spp., *Colletotrichum* spp. (Filamentous fungi)	Recalcitrant compound degradation (lignin); humification; N stabilization (nitrification); N_2_ fixation; detoxification; stabilization.	[11,19,27,53,56,57]
**Transversal (All phases)**	Variable	Pseudomonadota (Proteobacteria)	Viral communities (varying by phase)	Organic matter transformation; internal nutrient recycling; stabilization of dissolved organic matter.	[43,54,58]

**Table 2:** Biotechnological strategies for nitrogen management during composting.

Category	Intervention	Feedstock and Conditions	Main Mechanism	Effect on N Dynamics	Key Outcomes	References
**Microbial Inoculation**	Functional consortia (Actinomycetes, Lactic Acid Bacteria, yeasts)	General organic waste	Enhances enzymatic regulation of ammonification and nitrification pathways	Promotes NH_4_^+^ transformation and N retention	Reduced NH_3_ emissions, improved compost quality	[17,44]
	*Bacillus* spp.–*Acinetobacter* spp. –*Lactobacillus* spp. consortia	Pig manure	Alters microbial succession; extends thermophilic phase.	Reduces gaseous N losses via mineralization control.	Increased stability and N conservation.	[81,82]
	Ammonia-oxidizing bacteria (AOB) Inoculants	Cattle manure	Sustains nitrification under high temperatures.	Enhances oxidation of NH_4_^+^ to NO_3_^−^	Enhanced N stabilization.	[53]
**Structural Additives**	Biochar + Magnesium	Livestock manure	Co-optimizes NH_3_ mitigation and heavy metal passivation in livestock manure. Provides microhabitats and adsorbs NH_4_^+^	Reduces NH_3_ volatilization through NH_4_^+^ adsorption and pH buffering	Reduced volatilization, Improved N retention and microbial stability	[77,84,85]
	Zeolite/Medical Stone	Pig manure	Physical adsorption and pH buffering.	Reduces NH_3_ volatilization through NH_4_^+^ adsorption and pH buffering	N losses: ~40% reduction; N_2_O: 36–69%	[20,80]
	Semi-coke	Dairy manure	Porous bulking agent; improves aeration.	Enhances nitrification capacity and supports AOB.	Improved nitrogen conservation.	[86]
	Coral sand	Tropical islands/Green waste	Alternative to vermiculite; increases aeration.	Reduces nitrogen loss during ventilation phases.	Enhanced nitrogen retention and compost stability.	[11]
	FeONPs (α-Fe_2_O_3_)	Agricultural waste	Selective inhibition of AOB over Ammonia-Oxidizing Archaea (AOA).	Inhibits excessive ammonification.	High NH_4_^+^-N reservation; reduced TN loss.	[87]
	Selenite [Se(IV)]	Goat manure + Straw	pH buffering; promotion of nitrification.	Regulates N_2_O and NH_3_ emission pathways.	NH3: 3.5–42.4% reduction; N_2_O: up to 69.54%	[83]
	Clinoptilolite	Chicken slurry + Rice straw	High Cation Exchange Capacity (100 cmol_c_\kg^−1^).	Absorption and adsorption of NH_4_^+^ and NO_3_^-^ into the lattice.	Reduced N loss; increased soil total N.	[80]
**Chemical Additives**	K_2_S_2_O_8_/S + MgSO_4_	Cow dung + Corn straw	Acidification; struvite formation.	Stabilizes N in mineral and crystalline forms.	Reduced volatilization.	[84,88]
**Organic Additives**	Wood/Bamboo vinegar	Pig manure	pH modulation; microbial niche alteration.	Inhibits NH_3_ release; stimulates nitrifiers.	Stimulates nitrifying community activity.	[79]
**Operational Control**	Aeration optimization (Free Air Space (FAS) adjustment)	Kitchen waste (45–65% FAS)	Enrichment of nitrifying genera (*Roseiflexus*, *Steroidobacter*) and inhibition of NH_3_ producing thermophiles (*Thermobifida*).	Enhanced oxygen diffusion and NH_4_^+^ oxidation to NO_3_^−^.	32% reduction in NH_3_ emissions; optimized compost maturity.	[81]

**Table 4:** Microbiome-based and physicochemical strategies for modulating phosphorus bioavailability and stabilization during composting.

Strategy Category	Agents/Additives	Primary Functional Objective	Microbial Mechanisms and Outcomes	Key References
Microbial Inoculation (Phosphate-Solubilizing Bacteria and Fungi)	*B. subtilis*, *Pseudomonas* spp., *Aspergillus* spp., *Penicillium* spp.	Increase P bioavailability	Solubilization: Increases available Pi (Olsen-P) through organic acid secretion and enzymatic mineralization of Po (e.g., phytates).	[49,90,91]
Microbial Inoculation Phosphate-Accumulating Bacteria (PAB)	*Halocella* sp.	P stabilization/Reduced P losses	Controlled Release: Absorbs excess phosphate and stores it intracellularly as Poly-P; increases phosphorus microbial biomass.	[92]
Carbon-based Amendments	Biochar	Increase P bioavailability/Reduced P losses	Habitat and Dissolution: Promotes PSB colonization, enhances dissolution of Ca-P and occluded P, and favors Pi redistribution toward bioavailable pools.	[101]
	Labile Carbon (e.g., Glucose)	P stabilization	Humification: Stimulates acid/alkaline phosphomonoesterases (AcP/AlP) and favors P sequestration into stable humic substances.	[49,76]
Mineral and organic Additives	Rock Phosphate, Thermophosphates and Phosphogypsum + PSB	Increase P bioavailability	Enrichment: Increases total and water/citrate-soluble P; improves material porosity and prolongs the thermophilic phase; Activation of P via genes *phoD* and *appA* in *Lactobacillus* sp. and *Pseudomonas* sp. in livestock and poultry manure.	[22,26,84,102]
	Wood Vinegar and Humic Acids	P stabilization/Reduced P losses	Struvite Formation: Acidifies the substrate to regulate P speciation and promotes Magnesium Ammonium Phosphate (struvite) formation.	[90]
Biological Pretreatment	Black Soldier Fly Larvae	Increase P bioavailability	Accelerated Maturation: Doubles total/available P through larval gut enzymes (phosphomonoesterases) and enrichment of native PSB.	[103,104]
Emerging Technologies	Microbial Fuel Cell (MFC)	P stabilization/Reduced P losses	Immobilization: Promotes humification and converts leachable exchangeable P into stable mineral forms (Al-P and Ca-P); stimulates C-P lyase and phytase activity.	[43]

## Data Availability

Data is contained within the review.

## Abbreviations

The following abbreviations are used in this manuscript:

Al-P	Aluminum-bound Phosphorus
AOB	Ammonia-oxidizing bacteria
AOA	Ammonia-Oxidizing Archaea
C	Carbon
Ca-P	Calcium-bound Phosphorus
Fe-P	Iron-bound Phosphorus
N_2_	Dinitrogen
MFC	Microbial Fuel Cell
NH_3_	Ammonia
NH_4_^+^	Ammonium
NO_3_^−^	Nitrate
NO	Nitric Oxide
NO_2_	Nitrite
N	Nitrogen
N_2_O	Nitrous Oxide
P	Phosphorus
Pi	Inorganic P
Po	Organic P
PAB	Phosphate-Accumulating Bacteria
Poly-P	Polyphosphates
PSB	Phosphate-Solubilizing Bacteria
sp.	means species, singular
spp.	means species, plural
WHC	water holding capacity
